# Investigation of Microenvironmental Exposures to Particle-Bound Polycyclic Aromatic Hydrocarbons for Elementary School Children

**DOI:** 10.3390/ijerph16224390

**Published:** 2019-11-10

**Authors:** Chin-Sheng Tang, Shih-Chun Candice Lung, Ta-Yuan Chang, Han-Hsiang Tu, Li-Te Chang

**Affiliations:** 1 Department of Public Health, College of Medicine, Fu Jen Catholic University, New Taipei City 242, Taiwan; tangcs@mail.fju.edu.tw (C.-S.T.); dndploneer@gmail.com (H.-H.T.); 2 Research Center for Environmental Changes, Academia Sinica, Taipei 11529, Taiwan; sclung@rcec.sinica.edu.tw; 3 Department of Occupational Safety and Health, College of Public Health, China Medical University, Taichung 404, Taiwan; tychang@mail.cmu.edu.tw; 4 Department of Environmental Engineering and Science, Feng Chia University, Taichung 407, Taiwan

**Keywords:** particle-bound polycyclic aromatic hydrocarbons, microenvironmental exposure, elementary school children, benzo[a]pyrene equivalent concentration, risk assessment

## Abstract

Polycyclic aromatic hydrocarbons (PAHs) are formed when organic matters incompletely combust and get distributed into the air in the form of vapor or the particular phase of absorption or condensation on the surface of respirable particles. Certain PAHs are considered as carcinogenic and mutagenic, and are primarily associated with the particulate phase. Therefore, the characterization of exposure to particle-bound PAHs (p-PAHs) is critical to assessing the health risks in our daily life. A panel study was conducted during the years 2004 and 2005 to assess microenvironmental exposures to p-PAHs for elementary school children living in Taipei metropolitan area. During the study, integrated filter samples were collected by a dust monitor (model 1.108, Grimm) for 17 p-PAH species analysis using gas chromatography with mass spectrometry (GC/MS). The sampling durations were five days. Overall, 52 samples for children’s microenvironmental exposures were included in the data analysis. Results showed that geometric mean (GM) levels (and geometric standard deviation) of p-PAH exposures were 4.443 (3.395) ng/m^3^ for children. The top three highest proportions of p-PAH components were indeno[1,2,3-cd]pyrene (IND) (21.7%), benzo[g,h,i]perylene (BghiP) (18.5%), and dibenz[a,h]anthracene (DBA) (9.1%), all of which are 5- or 6-ring p-PAHs. In addition, results from diagnostic ratios and principal component analysis (PCA) found that traffic pollution, incense burning, and cooking emission were the major p-PAH exposure sources for children. The total benzo[a]pyrene equivalent (BaPeq) concentration was 1.07 ± 0.80 ng/m^3^ (mean ± standard deviation), with a GM of 0.84 ng/m^3^. The GM value of the inhalation carcinogenic risk was 7.31 × 10^−5^ with the range of 2.23 × 10^−5^ to 3.11 × 10^−4^, which was higher than the U.S. Environmental Protection Administration guideline limit of 10^−6^. DBA accounted for 45.1% of the excess cancer risk, followed by benzo[a]pyrene (BaP) (33.5%) and IND (10.7%). In conclusion, the current study demonstrated that inhalational cancer risk due to the p-PAH exposures for children is not negligible, and more efficient technical and management policies should be adopted to reduce the PAH pollutant sources.

## 1. Introduction

Among air pollutants, particulate matter (PM) has been an important topic of continuous concern and discussion for public health. Studies have shown that long-term exposure to PM_2.5_ (particulate matter with an aerodynamic diameter less than 2.5 μm) in metropolitan areas increases the risk of lung cancer, adverse respiratory outcomes, and cardiovascular diseases [1,2,3]. Short-term exposure may cause symptoms of exacerbations in patients with respiratory diseases, including bronchial inflammation and the occurrence of asthma, and will change the variability of the patient’s heart rate [4,5]. In addition to the attention to the health hazards of particles with different sizes, the aerosol constituents also raise a serious concern in the scientific community [6]. Among them, polycyclic aromatic hydrocarbons (PAHs) are a group of persistent organic pollutants that have significant adverse effects on the health of the human body [7].

PAHs are hydrocarbons bonded by two or more fused aromatic rings, which are formed mainly as a result of incomplete combustion and pyrolysis of organic substances. The sources of PAHs can be classified as natural or anthropogenic. The PAHs in nature comes from natural combustion activities such as forest fires or volcanic eruptions, where there is a much lower amount of PAHs produced than that of human activities [8]. The sources of PAHs generated by human activities include mobile and stationary pollution sources. The mobile-oriented PAHs are mainly emitted by motor vehicles, while the stationary pollution sources include industrial process combustion, waste incineration, thermal power generation, and cooking activities. In addition, the geological factor, weather condition, urban land use characteristics, and customs of the various regions have different degrees of influence on the concentration of PAHs in the environment [9,10,11].

The most noticeable aspect of PAHs is their mutagenicity and carcinogenicity. Because of the relatively high vapor pressure and low molecular weight, lower-ring PAHs are often present in the atmosphere in a gaseous phase and are easily diluted by the atmosphere due to meteorological conditions. High molecular weight PAHs are mostly in the form of granular phase and are easy to adhere to particles. Previous studies have shown that particle-bound PAHs (p-PAHs) are absorbed predominantly on fine particulate matters [12,13,14]. It is highly toxic, and once inhaled into the human body, it may be converted into mutagenic substances or carcinogens due to the physiological metabolism of the human body, causing adverse health effects such as lung cancer and embryo mutation. Among the various PAH compounds, benzo[a]pyrene (BaP) has been identified by the International Agency for Research on Cancer (IARC) as carcinogenic to humans (Group 1) [15]. This compound is often used as an indicator of PAHs or as an indicator of air pollution carcinogenicity index, to explore the relative hazards of each PAHs species, or to calculate the BaP equivalent (BaPeq) concentration as a basis for assessing exposure and health risks.

Taiwan belongs to tropical and subtropical climate zones, with a relatively warm and humid climate throughout the year. In the summer, residents often turn on air-conditioners and close the windows, while there is no severe cold in winter. Compared to mid/high latitudes, people in Taiwan rarely use kerosene or wood burning to warm up, and the habits of cooking and worshipping in Taiwan are different from those in other countries. Therefore, it is important to assess personal exposure and health risks of PAHs in subtropical areas. Relevant research has been conducted on the effects of PAH pollution sources such as burning behaviors, cooking, or transportation on indoor concentrations [16,17,18]. However, according to the literature review, no studies have evaluated the exposure and carcinogenic risks of PAHs in an individual’s living microenvironment for susceptible subpopulations. This study aimed to measure microenvironmental p-PAH levels for elementary school children in the Taipei metropolitan area, where the results would serve to assess the exposure intensity and cancer risk of p-PAHs and to analyze the contribution of each source of exposure.

## 2. Materials and Methods

### 2.1. Study Design and Subject Characteristics

This study recruited elementary school children living in Xinzhuang District, New Taipei City, Taiwan to conduct a 5-day microenvironmental p-PAH sampling. As part of the project for adverse effects of PM pollution on New Taipei City residents, a detailed description of the study design has been reported previously [19]. A total of 43 children participated in the study, and each of them was monitored for 1 to 2 times (52 samples in total). The monitoring period of the study was from 24 March 2004 to 15 July 2005. At the beginning of each sampling session, the field staff obtained participants’ household information including the floor, house type, home area, and the material of the indoor compartment. During the monitoring, all subjects were instructed to keep the portable monitoring equipment with them at all times. When the subjects were out, a field staff member carrying the portable equipment was assigned to each subject from 7:00 a.m. to 9:00 p.m. daily to measure the participants’ p-PAH exposures during normal daytime activities. Detailed information on household activities and time-activity patterns of the monitored participant was noted in personal diaries. During each day, subjects reported various microenvironments they visited, including living room, bedroom, classroom, other non-residential indoor areas, and outdoor places. The information of diary entries was re-confirmed by the field staff on a daily basis. Microenvironmental p-PAH exposures were only counted if the monitor was with the subject at least 75% of the time during sampling. In fact, none of the participants reported time periods when the measurement devices were not with them. The review board of the Environmental Protection Department of New Taipei City approved the research protocol, and a written consent was obtained from each participant’s parents before the study was launched.

In the current study, most of the subjects lived near the local elementary school area, with 53% (23) of boys and 47% (20) of girls, the average age of them being 9 years (9.1 ± 1.8) and ranging from 6–11 years. With regards to the type of housing, 16% (7) of the participants lived in a single-family house, and 84% (36) of them lived in an apartment. In addition, according to the pollution source records of the time-activity diary, 25% (13) of the collected 52 samples were ever exposed to environmental tobacco smoke (ETS), 29% (15) had exposure to incense burning, and 54% (28) had cooking exposure.

### 2.2. Instrumental Measurement and Laboratory Analysis

In this study, a portable aerosol analyzer (Model 1.108, Grimm Aerosol Technik GmbH & Co. KG, Ainring, Germany) was used with Teflon filter for p-PAH sampling at the flow rate of 1.2 L/min. Teflon filters were weighed before and after sampling, under the conditions of temperature of 20–23 °C and relative humidity of 40–45% for 48-hour conditioning. The conditioned filter was weighed using a six-digit microbalance (Mettler Model MX5, Mettler Toledo International Inc., Greifensee, Switzerland), and a static eliminator was used to eliminate static electricity. Each piece of the filters was weighed twice. If the difference between the first and second weight was greater than 5 µg, it would be re-weighed. After the weighing was completed, the sampled filters were sealed and placed in the refrigerator for subsequent component analysis.

The weighted filters were subjected to qualitative and quantitative analysis by gas chromatography and mass spectrometer (GC/MS) (GC: Varian CP-3800; MS: Varian Saturn 2200, Palo Alto, CA, USA). The carrier gas of the gas chromatograph was nitrogen, using constant current mode analysis. The column used was Varian’s VF-5ms column, and the initial and end temperatures of the oven were 60 °C and 300 °C, respectively. In the pre-treatment part of the sample, the filter sample was first added to the recovery standard, and the mixture was extracted three times with a mixture of n-hexane and dichloromethane, and then the extract was concentrated with nitrogen. The sample was then purified by self-filling the rubber tube column, and finally the purified extract was concentrated to 0.2 mL, then added the internal standardized elements for qualitative and quantitative analysis. A total of 17 p-PAHs were analyzed in this study, including 3-ring compounds of acenaphthylene (Acy), acenaphthene (Ace), fluorene (Flu), phenanthrene (Phen) and anthracene (Ant), 4 rings of fluoranthene (Flrt), pyrene (Pyr), benz[a]anthracene (BaA) and chrysene (Chry), and higher-ring compounds (5 and 6 rings) of benzo[b]fluoranthene (BbF), benzo[k]fluoranthene (BkF), benzo[e]pyrene (BeP), BaP, perylene (Per), indeno [1,2,3-cd]pyrene (IND), dibenz[a,h]anthracene (DBA), and benzo[g,h,i]perylene (BghiP). All p-PAH analysis data were corrected by laboratory blanks. The average recovery rate of 17 p-PAHs was 87.3%. The recovery rates and method detection limits of each p-PAHs were listed in Table 1.

### 2.3. Data Processing and Statistical Analysis

To ensure the quality of data processing, all original records were stored in Excel 2003 files (Manufacturer, City, US State abbrev. if applicable, Country) for verification by double entry. In addition to descriptive statistical analysis, this study used the Mann–Whitney U test to examine if the levels of p-PAHs differ by the observed sources in a microenvironment, and employed the diagnostic ratios and principal component analysis (PCA) to investigate the contribution of the sources to microenvironmental exposures. Statistical significance was inferred at a *p*-value of 0.05 in the study. The statistical analysis software used was SPSS 12.0 (SPSS Inc., Chicago, IL, USA).

While carrying out the PCA, the KMO (Kaiser–Meyer–Olkin) sampling adequacy test and the Bartlett ball type test were adopted to confirm the distribution characteristics of the collected data. If the KMO value was less than 0.5, the PCA would not be suitable, whereas the larger the value of the chi-square of the Bartlett ball test, the more suitable it would be. The KMO value in the study was 0.551, and the Bartlett ball type test was significant, indicating that PCA was suitable. The rotation method used in the PCA was the maximum variation method (varimax). According to the guidance proposed by Hopke et al., the factor with eigenvalue greater than 1 is the main contributor, and then the factor loading serves as the selection criterion [20]. When the factor loading is greater than 0.7, it is the main species, and if it is between 0.5 and 0.7, it is the secondary species. Since the secondary species only had moderate loadings, the results of the study would be interpreted by the main species.

Finally, based on the concept of toxic equivalency factor (TEF), this study converted the exposure concentrations of each p-PAHs into BaP equivalent (BaPeq) concentrations and calculated the cancer risk via inhalational exposure. The TEFs used in this study had been compiled by Tsai et al., where the BaP equivalent coefficients for 17 p-PAHs were Acy of 0.001, Ace of 0.001, Flu of 0.001, Phen of 0.001, Ant of 0.01, Flrt of 0.001, Pyr of 0.001, BaA of 0.1, Chry of 0.01, BbF of 0.1, BkF of 0.1, BeP of 0.01, BaP of 1, Per of 0.001, IND of 0.1, DBA of 1, and BghiP of 0.01 [21]. The 17 p-PAH concentrations were multiplied by the corresponding equivalency factors and then summed to obtain the total BaPeq (in ng/m^3^). The lifetime excess cancer risk (LECR) via inhalational exposure to p-PAHs was determined as the multiplication result of the total BaPeq and World Health Organization (WHO) unit risk of 8.7 × 10^−5^ ((ng/m^3^)^−1^) [22].

## 3. Results and Discussion

### 3.1. Descriptive Analyses for p-PAHs Exposures

Table 2 showed the descriptive results of the microenvironmental p-PAHs exposures for the children, with the geometric mean (GM) concentration of total p-PAHs of 4.443 ng/m^3^, the geometric standard deviation (GSD) of 3.395 ng/m^3^, and the range of 1.292–17.468 ng/m^3^. It could be seen that the exposure levels for p-PAHs with lesser rings (below 5 rings) were quite low. In addition, by comparing the ratio of the exposure concentration of each p-PAHs species divided by the exposures concentration of total p-PAHs, the top three major compounds of the children’s microenvironmental p-PAHs exposures were IND (21.7%), BghiP (18.5%), and DBA (9.1%), all of which were 5- or 6-ring p-PAHs.

Basically, the total p-PAH exposure concentrations in this study were similar in magnitude to those of other studies, although the PAH species analyzed in each study were not necessarily identical. Ohura et al. surveyed personal, residential, workplace, and outdoor microenvironments for 55 adults in Japan, analyzing 21 p-PAH species for samples taken in summer and winter. The median personal exposures were 3.70 and 3.69 ng/m^3^, respectively, which was comparable to the median exposure concentration of the current study (4.127 ng/m^3^) [23]. For the study of indoor p-PAH contaminants, Levy et al. conducted p-PAHs sampling in six indoor microenvironments (library, coffee shop, shopping mall, restaurant, apartment, and hospital) in Boston, MA, USA [24]. The p-PAH range was 5–12 ng/m^3^, which was also comparable to the exposure of the current study. For the study in urban areas with different climates and different types of emission sources, Naumova et al. reported that the total PAH concentrations in indoor samples were 16–220 ng/m^3^ in Los Angeles, CA, USA, 21–310 ng/m^3^ in Houston, TX, USA, and 22–350 ng/m^3^ in Elizabeth, NJ, USA, whose concentration ranges were higher than the variability of the current study, presumably related to the indoor activity characteristics [25].

### 3.2. Analysis of p-PAHs Exposures Based on Univariate Analysis and Diagnostic Ratios

By means of the information on the potential pollution sources collected in the time-activity diary, the non-parametric Mann–Whitney U test was adopted to examine if the microenvironmental concentrations of p-PAHs were associated with the presence of certain sources indicating exposure. (Table 3). Results showed that there was a statistically significant difference for ETS exposure, with the higher exposure levels of IND and BghiP found in the ETS-exposed group. There were also statistically significant differences in Flu, Phen, Pyr, BaA, Chry, and total p-PAHs under the exposure to incense burning. Past studies have found that the main p-PAH species emitted by incense sticks were Flu, Phen, Pyr, BaP, and BghiP [26,27,28,29]. Findings of the current study, the statistically significant differences of the p-PAHs (namely Flu, Phen, and Pyr) concentrations for incense-exposure groups, are consistent with the findings from previous studies. On the other hand, no statistically significant differences of p-PAHs for cooking-exposure groups were reported, presumably because the collected p-PAH samples had more than one pollution source.

To characterize the PAHs emission sources, previous studies have found that there were specific dominant PAHs emission species for traffic and industrial sources. The diagnostic ratio, which is based on the relative abundance of individual PAHs or groups of PAHs, has been widely used to identify sources of PAHs in diverse environments [30,31]. To avoid the bias caused by the extreme values, we used the interquartile range (IQR) to explore the range of diagnostic ratios for children’s microenvironmental p-PAHs exposures. In the current study, the diagnostic ratios of BaA/BaP, BaA/Chry, BaP/BghiP, BghiP/BaP, IND/BghiP, BghiP/IND, Flrt/Pyr, BaP/(BaP+Chry), FL/(FL+Pyr), and IND/(IND+BghiP) ranged between 0.10–0.33, 0.76–2.00, 0.18–0.47, 1.88–3.14, 0.98–1.37, 0.73–1.03, 0.71–1.63, 0.76–0.91, 0.19–0.36, and 0.49–0.58, respectively. Compared with the typical diagnostic ratios for traffic and industrial PAH sources published in the literature (Table 4), results of eight diagnostic ratios, including BaA/Chry, BaP/BghiP, IND/BghiP, BghiP/IND, Flrt/Pyr, BaP/(BaP+Chry), FL/(FL+Pyr), and IND/(IND+BghiP), all suggested gasoline and diesel exhaust sources, indicating that the microenvironmental p-PAH exposures for elementary school children were influenced by traffic emission [32,33,34,35,36,37,38,39,40].

### 3.3. Principal Component Analysis for p-PAH Exposure Sources

Due to the limitation of PAH diagnostic ratios in the emission source identification, PCA has been applied as a multivariate statistical tool to identify the major sources for air pollutants [13,41]. Table 5 presented the factor loadings of five principal components (PC) that accounted for microenvironmental p-PAH exposures of children, with 78.09% of total variance explained. PC1 through PC3, respectively, explained 25.54%, 23.90%, and 11.72% of the total variance. PC1 had high loadings for BaP, BbF, BghiP, BeP, and IND, which were characteristic of traffic emission [42,43]. PC2 showed high loadings for Pry, Flu, and DBA, which were related to incense burning [28,29,44]. PC3, with high loadings of Chy and BaA, corresponded to cooking sources [45,46]. PC4 contributed 9.38% of the p-PAHs data with the high loadings for Ant and Phen, while PC5 included Ace and Per with the explained variation of 7.55%. Both PCs were not found to be the indicators of specific emission sources in the literature.

Compared to the findings of previous local studies, Yang et al. investigated PM_2.5_-bound PAHs to determine the seasonal changes in total BaPeq concentrations and to identify PAHs sources in Hsinchu City, northern Taiwan. The two major sources were stationary emission sources (56.2%) and un-burned petroleum/traffic emissions (34.1%), which together accounted for 90.3% of PM_2.5_-bound PAHs [11]. For the study of a rural residential area nearby an oil refinery and petrochemical complex in Changhua County, central Taiwan, potential sources of PM_2.5_-bound PAHs were found to include un-burned petroleum/traffic emissions (42%), steel industry and coal combustion (31%), and petroleum and oil burning (27%) [10]. Contributing factors of p-PAHs from the aforementioned studies differed from findings in the current study, presumably because those measurements were only made for ambient environments. On the other hand, Li et al. evaluated the indoor and outdoor PAH concentrations in both summer and winter seasons in the urban atmosphere of Taipei areas, the results of which showed that there were three, four, and five factors accounting for 60, 75, and 68% of the variances for all homes, incensed homes, and non-incensed homes, respectively [27]. For incensed homes, PAHs could be attributed mainly (40% of the variance) to incense burning, while background sources might be the largest contributor to PAHs in non-incensed homes. The explained variance of Li’s research in Taipei was comparable to those in the current study. Overall, based on the results of diagnostic ratios and PCA, the main contributing factor of microenvironmental p-PAH exposures for elementary school children in the study was traffic pollution, while incense burning and cooking emission also had certain influence.

### 3.4. BaPeq Concentratons and Inhalation Cancer Risk Assessment

**Figure 1:**
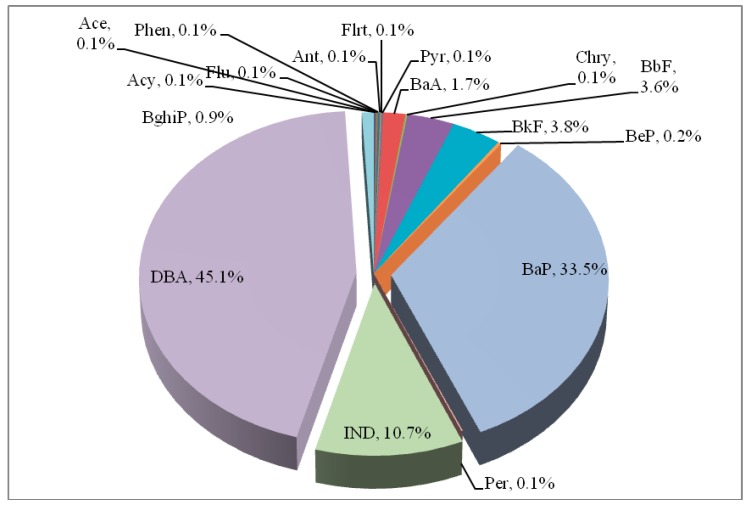
Proportions of BaPeq among the p-PAH components.

The toxicity equivalent concentration has widely used to assess risk of carcinogenic potency of PAHs [21,47,48]. In the current study, the total BaPeq concentration was 1.07 ± 0.80 ng/m^3^ (mean ± standard deviation), with a GM of 0.84 ng/m^3^ (data not shown). Based on the estimated total BaPeq, the GM of the inhalation carcinogenic risk for elementary school children was 7.31 × 10^−5^ (7.31 additional cases per 10,000 people exposed) with the range of 2.23 × 10^−5^ to 3.11 × 10^−4^, which was higher than the U.S. Environmental Protection Administration guideline limit of 10^−6^. Moreover, the relative contributions of individual p-PAHs to total BaPeq were shown in Figure 1, where the top three major species for BaPeq proportions were DBA, BaP, and IND, accounting for 45.1%, 33.5%, and 10.7%, respectively, of the total BaPeq in the particulate phase. Although BghiP had the 2nd-highest concentrations of the individual p-PAHs for children’s microenvironmental exposures (Table 2), it made a small contribution to the overall carcinogenic risk because of its low TEF.

Results for the BaPeq and risk assessment of the study could be further compared with findings from other local studies. Previous research had reported the LECRs in different areas of Taiwan. In Hsinchu City, the major BaPeq contributors were BaP, BbF, INP, and DBA, where BaP accounted for 49.0% of total BaPeq concentrations in all four seasons, and the annual average LECR of PM_2.5_-bound PAHs was 1.60 × 10^–5^ [11]. The means of the LECR resulting from inhalation exposure to ambient p-PAHs were recorded as 4.7 × 10^−5^ for a rural residential area in Changhua County, 5.79 × 10^−4^ for an urban trafficked area (southern Taiwan), 2.56 × 10^−4^ for a suburban area (central Taiwan), and 2.57 × 10^−5^ for a rural area (Yunlin County) [10,49,50,51]. Except for traffic emission, all of the above sampling sites were within a certain distance of petrochemical complex, industrial park, coal-fired power plant, or waste incinerator. On the other hand, as to occupational PAH exposures, Tsai et al. performed PAH sampling analysis for highway toll station workers. The results showed that the total BaPeq exposure levels for attendants in the three work-shifts (day, night, and late night) were 230, 203, and 151 ng/m^3^, respectively, which were higher by two orders of magnitude compared to the current study, this being due to tollbooth workers’ high exposures to traffic emission [21]. In addition, in the literature, median or mean ILCRs for workers included 2.96 × 10^−5^ in temple workers, 1.6 × 10^−5^ in motorcycle commuters, 2.3 × 10^−4^ in topside coke oven workers, and 3.14 × 10^−5^ for street food carts [46,52,53,54]. Overall, in comparison with other local studies, inhalation exposure to p-PAHs also posed a non-negligible cancer risk to the children in Taipei metropolitan area, indicating more efforts should be taken into account for environmental health protection.

### 3.5. Strength and Limitations of the Study

To our knowledge, this is the first to measure microenvironmental exposures to p-PAHs and to estimate the corresponding inhalational cancer risk for elementary school children. The estimation was more representative of human exposure compared to previous studies that have used area samples to assess the carcinogenic potency [55]. Our results further justified a need to implement strategies that may contribute to pollution mitigation and prevention and to provide healthier environments for children. Even so, the limitations of this study should be listed. First, considering the availability and feasibility of sampling equipments, the current study did not collect samples for gas-phase PAHs, which could underestimate the health risk associated with atmospheric PAHs. In fact, children might be exposed to PAHs through contaminated foods or skin contact, which also increase the cancer risk [56]. Moreover, although risk assessment is a power tool to provide risk information for environmental legislation, there are also areas of uncertainty in the case of PAHs that should be taken into account. One of the uncertainties is the TEF values, which were established from toxicological animal studies, and the other is the unit risk value of BaP, which was extrapolated from the results of epidemiological studies with high exposure concentrations. Some studies have pointed out that the risk estimates could be biased in some working environments [57,58]. Since the use of PM-bound BaP as the only marker of exposure to carcinogenic PAHs may not be enough, there is a clear need to revise the established international guideline by including other health-relevant PAHs rather than BaP alone. In addition, more studies including both environmental monitoring and children biomonitoring are needed to fully understand the health implications of PAHs in children [55].

## 4. Conclusions

In this study, microenvironmental p-PAHs for elementary school children in the Taipei metropolitan area were measured to evaluate the p-PAHs exposures, the contribution of potential exposure sources, and the inhalational carcinogenic risk. Results showed that the children’s microenvrionmental p-PAHs exposures were dominated by the high-ring species (IND, BghiP, and DBA from lowest to highest). Based on the diagnostic ratios and PCA, the main contributing factors of p-PAHs exposure in children included traffic pollution, incense burning, and cooking emission. Concerning the individual toxicity of the target p-PAHs, the compounds that contributed most to the total estimated risk were DBA (45.1%), BaP (33.5%), and IND (10.7%). Finally, the inhalation carcinogenic risk for children was moderately higher than 10^−6^, with a GM value of 7.31 × 10^−5^. In brief, our findings added to the growing evidence for the potential health effects of p-PAH exposures. Controlling the emissions sources is a priority for governmental agencies, and the combination of studying exposure, source apportionment, and health is recommended for future research.

## Figures and Tables

**Table 1:** Number of rings, molecular weight, recovery rates, and method detection limits for 17 p-PAHs.

Compound	Abbreviation	Number of Rings	Molecular Weight (g/mol)	Recovery (%) (Mean ± SD ^1^)	MDL ^2^ (pg/μL)
Acenaphthylene	Acy	3	152	77.4 ± 12.5%	5
Acenaphthene	Ace	3	154	74.4 ± 14.4%	5
Fluorene	Flu	3	166	78.5 ± 8.4%	5
Phenanthrene	Phen	3	178	82.6 ± 14.8%	5
Anthracene	Ant	3	178	74.0 ± 7.3%	5
Fluoranthene	Flrt	4	202	78.5 ± 8.4%	5
Pyrene	Pyr	4	202	90.3 ± 15.6%	5
Benz[a]anthracene	BaA	4	228	98.6 ± 21.1%	10
Chrysene	Chry	4	228	91.2 ± 22.6%	10
Benzo[b]fluoranthene	BbF	5	252	94.2 ± 9.3%	20
Benzo[k]fluoranthene	BkF	5	252	93.3 ± 24.2%	20
Benz[e]pyrene	BeP	5	252	95.1 ± 16.8%	20
Benzo[a]pyrene	BaP	5	252	92.0 ± 19.5%	20
Perylene	Per	5	252	91.8 ± 14.5%	20
Indeno[1,2,3-cd]pyrene	IND	6	276	94.1 ± 23.6%	10
Dibenz[a,h]anthracene	DBA	5	278	87.3 ± 18.8%	10
Benzo[ghi]perylene	BghiP	6	276	93.4±17.6%	10

^1^ SD: standard deviation. ^2^ MDL: method detection limit.

**Table 2:** Microenvironmental p-PAH exposures for elementary school children (ng/m^3^).

Compound	GM ^1^	GSD ^2^	Minimum	Median	Maximum
Acy	0.055	0.056	0.021	0.042	0.257
Ace	0.043	0.050	0.029	0.039	0.388
Flu	0.059	0.140	0.021	0.040	0.544
Phen	0.062	0.169	0.029	0.040	0.752
Ant	0.054	0.191	0.029	0.040	0.947
Flrt	0.096	0.250	0.029	0.052	0.944
Pyr	0.077	0.263	0.021	0.042	1.020
BaA	0.055	0.646	0.029	0.040	4.602
Chry	0.055	0.553	0.029	0.040	3.896
BbF	0.243	0.474	0.041	0.246	2.965
BkF	0.164	0.876	0.058	0.103	4.817
BeP	0.170	0.290	0.058	0.134	1.443
BaP	0.233	0.400	0.041	0.266	2.478
Per	0.103	0.180	0.058	0.081	0.843
IND	0.894	0.772	0.146	0.961	4.170
DBA	0.298	0.557	0.115	0.185	2.563
BghiP	0.786	0.587	0.082	0.821	2.285
Total p-PAHs ^3^	4.443	3.395	1.292	4.127	17.468

^1^ GM: geometric mean. ^2^ GSD: geometric standard deviation. ^3^ Total p-PAHs: summation of 17 p-PAHs

**Table 3:** Comparison of p-PAH concentrations for children exposed to potential pollution sources ^1^.

Compound	ETS ^2^			Incense burning		
	Non-Exposed ^3^ (*n* = 39)	Exposed ^3^ (*n* = 13)	*p*-Value	Non-Exposed ^3^ (*n* = 37)	Exposed ^3^ (*n* = 15)	*p*-Value
Acy	0.043	0.041	0.59	0.040	0.046	0.44
Ace	0.039	0.037	0.22	0.038	0.040	0.55
Flu	0.040	0.037	0.37	0.038	0.046	0.038*
Phen	0.041	0.039	0.63	0.039	0.054	0.023*
Ant	0.040	0.037	0.27	0.038	0.041	0.12
Flrt	0.050	0.104	0.73	0.045	0.175	0.06
Pyr	0.045	0.039	0.45	0.040	0.078	0.038*
BaA	0.043	0.037	0.37	0.038	0.058	0.013*
Chry	0.041	0.037	0.36	0.038	0.051	0.009*
BbF	0.182	0.305	0.26	0.218	0.437	0.37
BkF	0.099	0.159	0.89	0.098	0.159	0.10
BeP	0.108	0.237	0.05	0.111	0.292	0.16
BaP	0.237	0.367	0.27	0.237	0.407	0.18
Per	0.081	0.081	0.98	0.079	0.092	0.22
IND	0.864	1.672	0.003*	0.901	1.415	0.12
DBA	0.185	0.161	0.71	0.162	0.313	0.10
BghiP	0.738	1.516	0.002*	0.766	1.156	0.26
Total p-PAHs ^4^	3.312	6.241	0.06	3.312	7.163	0.009 *

^1^ Mann–Whitney U test. ^2^ ETS: environmental tobacco smoke. ^3^ median concentrations, ng/m^3^. ^4^ Total p-PAHs: summation of 17 p-PAHs. * Statistical significance.

**Table 4:** Some PAHs diagnostic ratios used for traffic and industrial source identification.

Diagnostic Ratio	Value	Pollution Source	Literature
BaA/BaP	0.5	Gasoline exhaust	32
	1.0	Diesel exhaust	
BaA/Chry	0.28–1.2	Gasoline exhaust	
	0.17–0.36	Diesel exhaust	
BaP/BghiP	1.25 1.7	Motor vehicle emissions Home heating	33
BaP/BghiP	0.30–0.78	Affected by the emission of motor vehicles, the greater the ratio, the greater the impact	34
BaP/BghiP	0.3–0.4 0.46–0.81	Gasoline exhaust Diesel exhaust	34
BaP/BghiP	>0.6	Motor vehicle exhaust, indicating a significant traffic flow	35
IND/BghiP	0.4 1.0	Gasoline exhaust Diesel exhaust	36
BghiP/BaP	1.16	Diesel exhaust	37
BghiP/IND	3.5 1.1	Gasoline exhaust Diesel exhaust	32
Flrt/Pyr	1	Motor vehicle	38
Flrt/Pyr	0.15 0.5 1.7 3 65	Urban incinerator Petroleum refinery Fertilizer plant Coal-fired power plant Steel mill	33
BaP/(BaP+CHR) Flrt/(Flrt+Pyr) IND/(IND+BghiP)	0.49 <0.5 0.18	Gasoline exhaust	39, 40
BaP/(BaP+CHR) Flrt/(Flrt+Pyr) IND/(IND+BghiP)	0.73 >0.5 0.35–0.70	Diesel exhaust	39, 40

**Table 5:** Eigenvalues, variance explained, and loadings of factors

PC *	Factor Loading	Eigenvalue	Explained Variance
**PC 1**		4.431	25.54%
BaP	0.902		
BbF	0.895		
BghiP	0.860		
BeP	0.849		
IND	0.808		
**PC 2**		4.064	23.90%
Pyr	0.933		
Flu	0.926		
DBA	0.861		
Flrt	0.667		
Acy	0.147		
**PC 3**		1.993	11.72%
Chy	0.964		
BaA	0.948		
BkF	0.560		
**PC 4**		1.595	9.38%
Ant	0.855		
Pnen	0.834		
**PC 5**		1.283	7.55%
Ace	0.835		
Per	0.800		

* PC: principal component.
